# The Meningitis and Encephalitis Registry of Lower Saxony, Germany (MERIN) – design and main results of circulating neurotropic pathogen surveillance, 2003 to 2023

**DOI:** 10.2807/1560-7917.ES.2026.31.6.2500625

**Published:** 2026-02-12

**Authors:** Mareike L Wollenweber, Konrad Beyrer, Armin Baillot, Masyar Monazahian, Elke Mertens, Sophie Rettenbacher-Riefler

**Affiliations:** 1 Public Health Agency of Lower Saxony, Hannover, Germany; 2 Postgraduate Training for Applied Epidemiology, Department for Infectious Disease Epidemiology, Robert Koch Institute, Berlin, Germany; 3 ECDC Fellowship Programme, Field Epidemiology path (EPIET), European Centre for Disease Prevention and Control (ECDC), Stockholm, Sweden

**Keywords:** enterovirus, surveillance, polio, viral meningitis, viral encephalitis, aseptic meningitis, acute flaccid paralysis

## Abstract

**BACKGROUND:**

The Meningitis and Encephalitis Registry in Lower Saxony (MERIN), introduced in 2003, monitors circulating neurotropic pathogens in Lower Saxony and Bremen and offers free laboratory diagnostics for patients hospitalised with aseptic meningitis, encephalitis and polio-like symptoms.

**AIM:**

We aimed to present set-up and operation of MERIN in detail and provide results of the collected data.

**METHODS:**

Data (work-flow, demographics, sample materials, detected pathogens including non-polio enterovirus (NPEV) genotypes), collected between 2003 and 2023 were extracted from the MERIN database and presented descriptively. Seasonal patterns of detected pathogens were analysed using a Poisson regression model.

**RESULTS:**

During 21 years of MERIN’s operation, 34,688 samples from 13,813 patients were analysed, 54.6% (7,548/13,813) of which were male. The majority of patients were children, with 58.8% (8,127/13,813) under the age of 10 years. Twenty different pathogens were identified; NPEV infections constituted 56.9% (2,372/4,172) of all diagnoses and were found in 17.2% of patients (2,372/13,813). *Borrelia burgdorferi* *sensu lato*, adenovirus and varicella-zoster virus were identified in 7.3% (1,004/13,813), 2.1% (286/13,813) and 1.4% (190/13,813) of patients, respectively. Most frequently occurring NPEV genotypes were echovirus 30 (n = 437), echovirus 6 (n = 223) and coxsackie B virus (n = 103). Polioviruses were not detected. Increased numbers of patients and detected pathogens during summer months resulted in seasonal peaks.

**CONCLUSION:**

MERIN elucidates the spectrum of circulating pathogens, mostly NPEV, causing symptoms of aseptic meningitis, encephalitis and polio-like symptoms and demonstrates seasonal occurrence of pathogens. MERIN contributes to the German national enterovirus surveillance and documents the polio-free status of Lower-Saxony and Bremen.

**Key public health message**

**What did you want to address in this study and why?**

When the reporting obligation for aseptic meningitis/encephalitis ended with implementation of the German Infection Protection Act in 2001, the Meningitis and Encephalitis Registry in Lower Saxony (MERIN), a voluntary surveillance system was implemented in the German federal state of Lower Saxony. We describe its set-up, operation and present some results from its 21 years of surveillance.

**What have we learnt from this study?**

MERIN monitors circulating neurotropic pathogens in hospitalised patients with aseptic meningitis, encephalitis and polio-like symptoms and ensures timely and reliable differential diagnostic clarification for patients of voluntarily participating hospitals. Our findings contribute to a greater understanding of pathogen circulation in general, with 20 different pathogens found. Non-polio enteroviruses constituted more than half of all detected pathogens, followed by *Borrelia burgdorferi sensu lato* and adenovirus. Our findings also support outbreak detection and management.

**What are the implications of your findings for public health?**

Since MERIN detected enteroviruses, but no polioviruses, it provides the required information and case numbers to document polio-free status of the German federal states of Lower-Saxony and Bremen and contributes to the national polio surveillance. By using additional clinical symptoms for admission, MERIN offers an alternative approach to acute flaccid paralysis surveillance in countries that have been polio-free for many years.

## Introduction

Infections of the central nervous system can result in meningitis, encephalitis or myelitis depending on the location of the inflammation (in the meninges, brain parenchyma or spinal cord), although combinations can occur [1-4]. Children and elderly people are at highest risk for complications and hospital admission rates are highest in infants [1,5,6]. In Europe, annual incidence of viral meningitis was estimated at 30 per 100,000 inhabitants in 2022 [3,5]. For encephalitis, annual incidence was estimated between 1.5 and 7.0 cases per 100,000 inhabitants [7].

Since meningitis and encephalitis cause a considerable burden of disease [2], under German federal law it was mandatory to notify bacterial and viral meningitis cases until 2001. The implementation of the German Infection Protection Act (IfSG) in 2001 [8] limited notification to bacteria causing septic meningitis (meningococcal meningitis) and ended reporting obligations for aseptic meningitis.

Aseptic meningitis and encephalitis are caused by pathogens other than pus-producing bacteria, mostly viruses. Across Europe, these pathogens most commonly include non-polio enteroviruses (NPEVs), herpesviruses such as herpes simplex virus (HSV), varicella-zoster virus (VZV), Epstein-Barr virus (EBV), cytomegalovirus (CMV), mumps virus, influenza virus and tick-borne encephalitis (TBE) virus [1,9], as well as *Borrelia burgdorferi* bacteria causing Lyme neuroborreliosis [10]. However, the aetiology of aseptic meningitis or encephalitis can only be identified in about one third of cases [9,11,12].

The monitoring of circulating neurotropic pathogens was the primary objective of the Public Health Agency of Lower Saxony (NLGA) when it established the Meningitis and Encephalitis Registry of Lower Saxony (MERIN) in 2003 [4,13]. A secondary objective was poliovirus surveillance, since an absence of polioviruses among detected enteroviruses (EV) documents polio-free status. These objectives remain unchanged.

The Meningitis and Encephalitis Registry of Lower Saxony is a passive voluntary surveillance system hosted by the NLGA devoted to monitoring circulating neurotropic pathogens. Submissions to MERIN are based on clinical symptoms. Patients hospitalised with clinical symptoms of aseptic meningitis, encephalitis or polio-like symptoms such as acute flaccid paralysis (AFP), whose samples are submitted to MERIN, are eligible for a broad diagnostic panel offered by the laboratory of the NLGA, covering pathogens that cause aseptic meningitis, encephalitis or AFP. The treating physician decides what sample materials of which patients to submit to MERIN. The samples are labelled MERIN-samples and submitted to the NLGA laboratory along with the MERIN submission form. Patient samples are then analysed at the NLGA laboratory; individual results are reported back to the treating physician and included in the MERIN database. Aggregated data on pathogens are collected by the NLGA and findings on NPEVs are forwarded to the national enterovirus surveillance (EVSurv) (an overview of the MERIN set-up is given in the flowchart in Supplement S1). Participating physicians receive rapid and reliable diagnostic clarification; acceptability and overall satisfaction with the performance of MERIN has been documented [14].

In this article, we present the set-up and operation of the MERIN surveillance system in detail and provide results of surveillance data collected over two decades, including patient demographics, investigated sample materials and detected pathogens, as well as NPEVs and their most frequent genotypes. We analyse and describe observed seasonal patterns and discuss the potential effects of non-pharmaceutical interventions during the COVID-19 pandemic.

## Material and methods

### Setting

The MERIN surveillance system was implemented in the German federal state of Lower Saxony in 2003 and extended to the federal state of Bremen in 2011. Lower Saxony hosts a population of ca 8 million inhabitants, whereas the federal state of Bremen has ca 700,000 inhabitants, consists of two cities and forms an enclave in Lower Saxony. Information on the federal public health system is given in Supplement S2. Hospitals and clinics with paediatric, neurologic and internal medicine wards are eligible for participation in MERIN. The number of voluntarily contributing hospitals has varied over the course of MERIN’s runtime since some hospitals or wards have been closed or merged with other clinics, and other institutions have joined or discontinued their participation (for details on the recruiting process, see Supplement S3).

### Laboratory diagnostics

If a patient displays aseptic meningitis, encephalitis or polio-like symptoms, the treating physician is invited to submit specimen samples to the NLGA laboratory for analysis. Sample material and number of samples per patient is determined by the treating physician. The NLGA laboratory analyses the samples from stool, cerebrospinal fluid (CSF), serum or occasionally respiratory swabs or urine for a range of neurotropic pathogens considered causative for meningitis, encephalitis or polio-like symptoms using several laboratory methods such as PCR, virus isolation in cell culture, typing and serological analysis. Sample results and accompanying data are stored in the NLGA laboratory information system. The data are anonymised then transferred to the MERIN database.

Since 2003, the NLGA laboratory analytical panel for MERIN samples comprises the following pathogens and methods: to exclude or confirm a potential HSV infection, analysis of CSF via PCR is prioritised. If a CSF sample arrives at the NLGA laboratory before 13:00 on a weekday, results are communicated back to the hospital before 16:00 on the same day; CSF and stool samples are screened via PCR for EV genetic material, including polioviruses, and cultivated to enable typing by serum neutralisation tests. Only one isolate per patient, preferably from CSF, is typed; respiratory swabs are screened via PCR for relevant respiratory pathogens including entero- and rhinoviruses, and if positive, cultivated. Cultivation is performed on four different cell lines susceptible to EV, according to the World Health Organization (WHO) standard protocols [15]; samples positive for NPEV are typed via serum neutralisation tests using two different pools of equine antisera, the Lim, Benyesh-Melnick (LBM) pool [16] obtained from the Serum Statens Institute, Denmark and the Dutch Rijksinstituut voor Volksgezondheid en Milieu (RIVM) enterovirus serotyping pool [17]. PCR-positive samples, where isolation of NPEVs on cell culture is not possible, and isolates where typing via serum neutralisation tests was unsuccessful, are sequenced at the National Reference Centre for Poliomyelitis and Enteroviruses at the Robert Koch-Institute (RKI) in Berlin, Germany [18].

Serological testing for Immunoglobulin (Ig)G and IgM antibodies against EV, measles virus, mumps virus, TBE virus and VZV is performed using commercially available enzyme immunoassays. If tick bites or neuroborreliosis symptoms are indicated on the sample submission form, serological assessment for *B. burgdorferi* *sensu lato* species is performed via enzyme immunoassay and confirmed via immunoblot test to differentiate Lyme neuroborreliosis from viral meningitis or encephalitis. Additional diagnostics for pathogens such as CMV or EBV via serological analysis and PCR are offered on request after consultation with medical doctors at the NLGA. The MERIN surveillance analytical panel has been adjusted and extended in the past. Adenovirus- and influenza virus-serology was discontinued in 2018. In 2023, targeted typing of NPEV isolates via next-generation sequencing (NGS) was introduced by the NLGA laboratory. Whole genome sequencing using short read technology (Ilumina, San Diego, United States (US)) and a subsequent basic local alignment search tool from the National Center for Biotechnology Information (NCBI) provides information on virus type.

Except for NGS, all laboratory procedures including sample management, laboratory analysis, generation of medical reports, documentation and data management have been accredited since 2005. Details on laboratory workflows are given in Supplement S4.

### Data collection and storage

The NLGA laboratory’s submission form accompanies the submitted specimen samples. The submission form includes: contact details of the submitting hospital, patients’ personal data (sex as a binary variable, birth date, post code), onset and type of clinical symptoms, patients’ vaccination status against measles, mumps, VZV and TBE and type and date of specimens collected. These data are stored in the NLGA’s laboratory information system. Laboratory results relevant to MERIN and corresponding personal data are anonymised, then transferred to the MERIN database (Access, Microsoft, Redmond, US). The diagnostic results in MERIN are evaluated by medical doctors at the NLGA in conjunction with the clinical symptoms provided on the submission form. These doctors aim to identify one pathogen as the most likely causative agent for the meningitis, encephalitis or AFP symptoms, irrespective of the type and number of submitted samples per patient. The final diagnostic outcome as well as its reliability, based on the diagnostic method used, is assessed via an in-house scoring system and recorded in the MERIN database.

### Data analysis

The diagnostic outcomes presented in this article were assessed as described above. Data analysis was performed at patient level and stratified by age, sex, pathogen and investigation date. Data collected between 2003 and 2023 were extracted from the MERIN database and analysed descriptively using R version 4.2.2 [19], RStudio version 2023.09.1 and Microsoft Excel version 2108. Chi-square tests were performed to assess changes in sex distribution during MERIN’s runtime.

A Poisson regression model was fitted to further analyse the overall pattern of detected pathogens and seasonality in patient numbers between 2007 and 2023. In the regression model, harmonic terms were incorporated to capture a potential annual seasonality. Further binary terms were added to reflect known outbreak years (2008, 2013) as well as the years of the COVID-19 pandemic (covering 2020 and 2021 by definition). The model fit was assessed using Akaike Information Criterion (AIC).

## Results

### Patient demographics

Between 2003 and 2023, a total of 13,813 patients hospitalised with symptoms of meningitis, encephalitis or polio-like symptoms were investigated (median 697 patients annually, range: 279–911), 54.6% of them were male (Table 1). Most patients (78.6%) were children under the age of 15 years (10,855/13,813), with 74.9% (8,127/10,855) under the age of 10 years and 47.7% (5,181/10,855) under the age of 5 years (overall median age: 7 years, range: 0–91 years, interquartile range (IQR): 12 years).

**Table 1:** Age and sex distribution of patients in the Meningitis and Encephalitis Registry in Lower Saxony (MERIN) hosted by the Public Health Agency of Lower Saxony (NLGA), Lower Saxony and Bremen^a^, Germany, 2003–2023 (n = 13,813 patients)

Age group (years)	Total	%	Male	%	Female	%	Missing	%
< 1	2,304	16.7	1,377	59.8	902	39.1	25	1.1
1–4	2,877	20.8	1,662	57.8	1,190	41.4	25	0.9
5–9	2,946	21.3	1,721	58.4	1,179	40.0	46	1.6
10–14	2,728	19.8	1,411	51.7	1,288	47.2	29	1.1
15–19	1,740	12.6	763	43.9	956	54.9	21	1.2
20–39	433	3.1	213	49.2	216	49.9	4	0.9
40–59	398	2.9	207	52.0	188	47.2	3	0.8
≥ 60	379	2.7	189	49.9	190	50.1	0	0.0
Unknown	8	0.1	5	62.5	3	37.5	0	0.0
Total	13,813	100	7,548	54.6	6,112	44.3	153	1.1

^a^ Data from Bremen were added to Lower-Saxony data from 2011 onwards.

In children under 10 years, 58.6% (4,760/8,127) were male. In the age group 15–19 years, 43.9% were male, whereas in the older age groups sex was more equally distributed (Table 1). Sex distribution did not change significantly over time (p > 0.05). The majority of patients were hospitalised in paediatric clinics (median: 92.8% of patients, range: 67.0–96.6%; IQR: 3.8%), 6.6% were hospitalised in neurological wards and 0.6% in internal medicine wards.

### Sample types and detected pathogens

During 21 years of surveillance, 34,688 samples from 13,813 patients were submitted to MERIN (median: 2 samples, range: 1–5). Cerebral spinal fluid samples represented 37.6% (13,032/34,688) of specimens, 33.8% (11,729/34,688) were from blood or serum and 25.0% (8,678/34,688) from stool. Throat swabs (1.8%, 633/34,688) and urine samples (1.8%, 616/34,688) were rarely submitted.

Laboratory analysis detected 20 different pathogens related to aseptic meningitis, encephalitis or polio-like symptoms in 4,172 patients (30.2%), whereas in 69.8% of patients, no pathogen was detected (Table 2). Non-polio enterovirus infections accounted for more than half (56.9%; n = 2,372) of the microbiological diagnoses. *Borrelia* spp. (24.1%), adenovirus (6.9%) and VZV (4.6%) were also frequently found. Polioviruses were not detected (Table 2).

**Table 2:** Pathogens detected in patient samples submitted to the Meningitis and Encephalitis Registry in Lower Saxony (MERIN) and analysed by the Public Health Agency of Lower Saxony laboratory, Lower Saxony and Bremen^a^, Germany, 2003–2023 (n = 13,813 patients)

Pathogens	n	% (n/13,813)	% of detected pathogens (n/4,172)
No pathogen detected	9,641	69.8	NA
Non-polio enterovirus (NPEV)	2,372	17.2	56.9
*Borrelia burgdorferi sensu lato*	1,004	7.3	24.1
Adenovirus	286	2.1	6.9
Varicella-zoster virus	190	1.4	4.6
Herpes simplex virus	166	1.2	4.0
Influenza virus	33	0.2	0.8
Cytomegalovirus	25	0.2	0.6
Epstein-Barr virus	21	0.2	0.5
Measles virus	20	0.1	0.5
Tick-borne encephalitis virus	20	0.1	0.5
Mumps virus	12	0.1	0.3
Respiratory syncytial virus	7	0.1	0.2
SARS-CoV-2 virus	5	< 0.1	0.1
Parainfluenza virus	3	< 0.1	0.1
Bocavirus	2	< 0.1	< 0.1
Metapneumovirus	2	< 0.1	< 0.1
Human herpes virus 6	1	< 0.1	< 0.1
John Cunningham virus	1	< 0.1	< 0.1
Rotavirus	1	< 0.1	< 0.1
Zika virus	1	< 0.1	< 0.1
Total	13,813	100.0	100.0

NA: not applicable; SARS-CoV-2: severe acute respiratory syndrome coronavirus 2.

^a^ Data from Bremen were added to Lower-Saxony data from 2011 onwards.

### Enterovirus genotypes

Subtyping was successful in 54.8% (1,300/2,372) of patients where NPEVs were determined to be the causative agent of meningitis, encephalitis or polio-like symptoms. Of the typable isolates, 92.9% (1,208/1,300) belonged to the enterovirus B species, of which isolates from five patients could not be further subtyped. A total of 27 different genotypes was identified, of which echovirus 30 (n = 437), echovirus 6 (n = 223) and coxsackievirus B5 (n = 85) were most prevalent (Table 3). In total, 103 isolates were identified as coxsackievirus B but not further differentiated. Seven percent of isolates (91/1,300) were identified as enterovirus A, of which 10 isolates were only assigned at species level. A total of eight different genotypes were detected among the enterovirus A isolates, with enterovirus A71 as the most prevalent genotype (n = 30), followed by coxsackievirus A2 (n = 17) and coxsackievirus A6 (n = 10). One isolate was identified as enterovirus C but could not be further subtyped. Enterovirus D was not found.

**Table 3:** Genotypes of non-polio enteroviruses in patient samples submitted to the Meningitis and Encephalitis Registry in Lower Saxony (MERIN) and analysed by the Public Health Agency of Lower Saxony laboratory, Lower Saxony and Bremen^a^, Germany, 2003–2023 (n = 1,300 patients)

Enterovirus A		Enterovirus B		Enterovirus C		Enterovirus D	
**Genotype**	**n**	**Genotype**	**n**	**Genotype**	**n**	**Genotype**	**n**
Not further subtyped	10	Not further subtyped	5	Not further subtyped	1	Not further subtyped	0
Enterovirus A71	30	Coxsackievirus B	103				
Coxsackievirus A2	17	Coxsackievirus B5	85				
Coxsackievirus A6	10	Coxsackievirus A9	46				
Coxsackievirus A4	7	Coxsackievirus B4	31				
Coxsackievirus A10	7	Coxsackievirus B3	16				
Coxsackievirus A16	5	Coxsackievirus B2	14				
Coxsackievirus A5	3	Coxsackievirus B1	8				
Coxsackievirus A8	2	Echovirus 30	437				
		Echovirus 6	223				
		Echovirus 11	60				
		Echovirus 9	45				
		Echovirus 25	29				
		Echovirus 18	19				
		Echovirus 7	18				
		Echovirus 13	14				
		Echovirus 3	11				
		Echovirus 4	9				
		Echovirus 14	9				
		Echovirus 5	6				
		Echovirus 2	4				
		Echovirus 15	4				
		Echovirus 21	4				
		Echovirus 33	3				
		Echovirus 17	2				
		Echovirus 1	1				
		Echovirus 20	1				
		Echovirus 29	1				
Total	91	Total	1,208	Total	1	Total	0

^a^ Data from Bremen were added to Lower-Saxony data from 2011 onwards.

### Pathogen detection over time

The number of patients and proportions of detected pathogens differed throughout the years studied. The number of annually investigated patients increased steadily between 2003 (n = 279) and 2008 (n = 720). Between 2009 and 2019, 600–900 patients were investigated each year. The highest number of investigated patients were seen in 2011 (n = 911), 2013 (n = 899) and 2016 (n = 870). In 2020, the number of patients investigated dropped to below 600 but have been increasing since.

Pathogens were identified in 30.2% of patients investigated (Table 4). The highest proportions were observed in 2007 (42.1%) and 2008 (41.7%), and the lowest in 2021, (17.4% of detected pathogens, 5.5% of NPEVs). Aside from the first year of MERIN (2003), where fewer patients were investigated, absolute numbers of pathogens were lowest during the COVID-19 pandemic years of 2020 and 2021.

**Table 4:** Yearly distribution of patient samples submitted to the Meningitis and Encephalitis Registry in Lower Saxony (MERIN), proportions of pathogens, non-polio enteroviruses and most prevalent non-polio enterovirus genotypes detected by the Public Health Agency of Lower Saxony laboratory, Lower Saxony and Bremen^a^, Germany 2003–2023 (n = 13,813 patients)

Year	Total patients	Pathogen positive patients		NPEV-positive patients		NPEV-positives in pathogen positive patients		Identified genotypes in NPEV-positive patients		Most prevalent genotype identified (if ≥ 10 EV typing results available)	Most prevalent genotype in NPEV-positive patients with identified genotype	
	n	n	%	n	%	n	%	n	%		n	%
2003	279	96	34.4	55	19.7	55/96	57.3	20	36.4	NA	NA	NA
2004	339	119	35.1	67	19.8	67/119	56.3	27	40.3	Coxsackievirus B	11	40.7
2005	470	171	36.4	104	22.1	104/171	60.8	59	56.7	Echovirus 30	28	47.5
2006	494	201	40.7	126	25.5	126/201	62.7	68	54.0	Echovirus 30 Coxsackievirus B	29 29	42.6 42.6
2007	516	217	42.1	128	24.8	128/217	59.0	16	12.5	NA	NA	NA
2008	720	300	41.7	194	26.9	194/300	64.7	148	76.3	Echovirus 30	133	89.9
2009	630	154	24.4	69	11.0	69/154	44.8	45	65.2	Echovirus 6	20	44.4
2010	663	209	31.5	114	17.2	114/209	54.5	61	53.5	Echovirus 30	18	29.5
2011	911	291	31.9	171	18.9	171/291	58.8	109	63.7	Echovirus 6	75	68.8
2012	744	173	23.3	107	14.4	107/173	61.8	79	73.8	Echovirus 11	23	29.1
2013	899	303	33.7	219	24.4	219/303	72.3	156	71.2	Echovirus 30	116	74.4
2014	766	190	24.8	94	12.3	94/190	49.5	54	57.4	Echovirus 30	10	18.5
2015	764	219	28.7	113	14.8	113/219	51.6	55	48.7	Echovirus 6	27	49.1
2016	870	300	34.5	160	18.4	160/300	53.3	66	41.3	Echovirus 30	14	21.2
2017	721	234	32.5	121	16.8	121/234	51.7	91	75.2	Echovirus 6	20	22.0
2018	714	221	30.9	124	17.4	124/221	56.1	91	73.4	Echovirus 30	37	40.7
2019	748	202	27.0	136	18.2	136/202	67.3	85	62.5	Coxsackievirus B5	20	23.5
2020	569	107	18.8	43	7.6	43/107	40.2	3	7.0	NA	NA	NA
2021	615	107	17.4	34	5.5	34/107	31.8	7	20.6	NA	NA	NA
2022	684	161	23.5	88	12.9	88/161	54.7	27	30.7	Coxsackievirus B	12	44.4
2023	697	197	28.3	105	15.1	105/197	53.3	33	31.4	Echovirus 11	10	30.3
Total	13,813	4,172	30.2	2,372	17.2	2,372/4,172	56.9	1,300	54.8	NA	NA	NA

EV: enterovirus; NPEV: non-polio enterovirus; NA: not applicable.

^a^ Data from Bremen were added to Lower-Saxony data from 2011 onwards.

Non-polio enteroviruses were detected in 17.2% of all patients investigated. The highest proportions of NPEV-positive patients in all investigated patients were seen in 2006, 2007, 2008 (25.5%, 24.8% and 26.9%, respectively) and 2013 (24.4%). The proportions of typable NPEVs varied considerably between years and reached 76.3% in 2008, 73.8% in 2012 and 75.2% in 2017 (Table 4).

The highest proportions of NPEVs in pathogen-positive patients were 64.7% in 2008 (194/300), 72.3% in 2013 (219/303) with echovirus 30 and 67.3% in 2019 (136/202) with coxsackievirus B5 as the most prevalent genotypes. Notably, in 2008, 89.9% of all typable isolates were identified as echovirus 30. In 2003 and 2007, no particular genotype was predominant. In 2020 and 2021, fewer than 10 isolates of one genotype were found.

In general, the proportion of detected pathogens was highest in children under 10 years of age. Most NPEV diagnoses were also found in this age group (75.7%; 1,795/2,372). In males, both proportions of detected pathogens and NPEVs were higher (60%) compared with in females (40%) (data not shown in table).

### Seasonality

Irrespective of annually varying patient numbers, more patients were investigated and more pathogens, including NPEVs, were detected between June and September compared to the rest of the year which resulted in consistent seasonal peaks (Figure).

**Figure:**
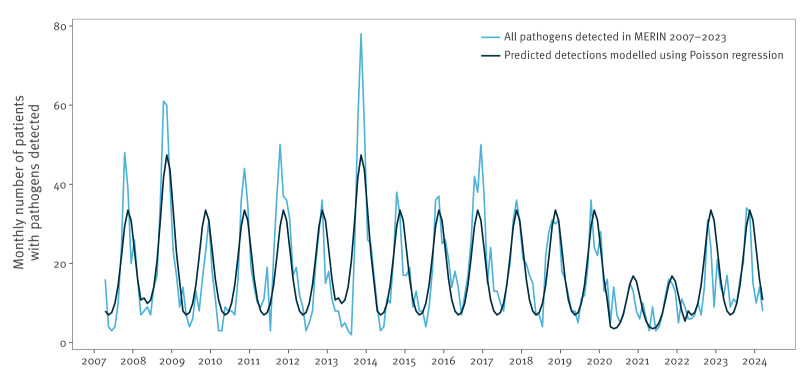
Pathogens detected in the Meningitis and Encephalitis Registry in Lower Saxony (MERIN) compared to predicted detections modelled using a Poisson regression with Akaike Information Criterion of 1,434.3, Lower Saxony and Bremen^a^, Germany, 2007–2023^b^

MERIN: Meningitis and Encephalitis Registry in Lower Saxony.

^a^ Data from Bremen were added to Lower-Saxony data from 2011 onwards.

^b^ The years 2003–2006 were not included in the Poisson regression model to avoid potential bias due to strong variation in patient numbers in the initial phase of the surveillance.

Pathogen data were analysed by the Public Health Agency of Lower Saxony laboratory.

The Poisson regression model yields statistical evidence for the observed seasonality (for details see Supplement S5), showing a consistent overall pattern in pathogen detection, except for individual outbreak years and the years of the COVID-19 pandemic (Figure). In 2020 and 2021, patient numbers as well as proportions of detected NPEVs were low and seasonal patterns were less pronounced (reduction by 49.7% for all detected pathogens and 67.0% for NPEVs; see Supplement S5). In 2022 and 2023, patient numbers returned to pre-pandemic levels.

## Discussion

The MERIN surveillance system targets patients hospitalised with symptoms of aseptic meningitis, encephalitis or polio-like symptoms from two German federal states, Lower Saxony and Bremen, which have a combined population of just under 9 million people. In the present investigation we characterise affected patient groups and provide an overview of detected pathogens investigated between 2003 and 2023.

Over the first 5 years of MERIN’s runtime, patient numbers increased continuously, reaching over 600 patients annually. Almost two thirds of patients who had specimens submitted to MERIN were under the age of 10 years and 37.5% were under 5 years. Young age is associated with increased incidence rates for hospitalisation with viral meningitis [6] due to higher risk of severe outcome [5] and higher susceptibility to NPEV infection [20], in particular of the central nervous system, presumably because the neuroimmune system has not yet fully developed [21]. Most patients with specimens submitted to MERIN were male; proportions of detected pathogens and diagnosed NPEV infections were also higher in males. Hospitalisation due to viral meningitis has been reported to occur more often in males than females [6] and higher incidences in young males have consistently been seen across countries [22] in EV surveillance systems [18,23] and NPEV outbreaks [24-26]. This may be due to the immune system reacting differently in males compared to females [27,28].

Cerebrospinal fluid samples were the primary investigated specimens in MERIN because a suspected brain inflammation is the main symptom for submitting patient samples to MERIN. As recommended by the European non-polio enterovirus network (ENPEN), participating physicians are encouraged to submit stool samples to increase EV detection [29,30].

According to the literature, the aetiology of ca 60% of all viral encephalitis and meningitis cases remains unexplained despite extensive testing [4,9,11], which corresponds with our findings of an infectious agent identified in only 30.2% (4,172/13,813) of patients. Non-polio enteroviruses were found in 56.9% (2,372/4,172) of all patient samples submitted to MERIN with pathogens detected. Other frequently observed viruses were adenovirus, VZV and the bacterium *B. burgdorferi s*.l. causing Lyme neuroborreliosis.

A review from 2017 found HSV, VZV and EV the most frequently detected pathogens causing aseptic infectious encephalitis [7]. The most common sources of aseptic meningitis in Europe are NPEVs, but HSV, TBE virus and VZV also contribute [9]. Data from MERIN are in agreement with these findings of the frequent occurrence of NPEVs and VZV. However, in over 21 years, HSV infection was only diagnosed in 1.2% (166/13,813) of patients with samples submitted to MERIN. In contrast to NPEV infections, which are often self-limiting, HSV encephalitis is highly lethal and thus requires immediate treatment [9].

Tick-borne encephalitis virus was rarely detected, which can be explained by the fact that it is not endemic in ticks in most parts of Lower Saxony and Bremen [31]. *Borrelia* spp. were the second most frequent pathogens found in MERIN. Lyme borreliosis is the most common tick-borne disease in Germany. In federal states with mandatory notification of Lyme borreliosis, cases peak during the summer months with more males affected than females [32].

In 2.1% (286/13,813) of investigated patients with aseptic meningitis, encephalitis or polio-like symptoms, adenovirus was found, often during virus isolation of stool samples. Adenoviruses typically cause mild infections of the gastrointestinal or respiratory tract, but infections can manifest as meningoencephalitis and have caused acute hepatitis outbreaks in children in 2021 and 2022 [33].

Comparisons of detection rates are hampered by different methodological setups. Enterovirus surveillance systems often receive pre-selected samples that have already tested positive for NPEV, resulting in positive proportions of 70–80% [18,23]. In syndromic systems that receive samples from general practitioners, proportions of detected pathogens can be lower than 10% [30]. One of the aims of MERIN is to diagnose and document the spectrum of pathogens causing aseptic meningitis, encephalitis or AFP symptoms. The extensive diagnostics offered ensure high acceptability of MERIN’s concept among treating physicians [14] but eventually decrease detection rates of NPEV.

In the present study, in 54.8% (1,300/2,372) of all patients diagnosed with an NPEV infection, a subtype could be assigned by either serotyping at the NLGA laboratory or genotyping at the German National Reference Centre for Poliomyelitis and Enteroviruses. Most typed isolates belonged to enterovirus A and B species, with the majority assigned to enterovirus B. These findings are similar to other European countries [34] and are reflected in EVSurv data [18]. In our analysis, echovirus 30 was the most frequently found genotype, which seemed to predominate every second or third year. Nationwide outbreaks of echovirus 30 occurred in 2008 [24] and 2013 [26] and an up-surge across Europe was observed in 2018 [35].

In 2011, echovirus 6 was prevalent in 68.8% (75/109) of all typable isolates. It was the second most frequent genotype registered in MERIN and was found to be circulating across Europe [34].

During years with reported outbreaks, higher patient numbers and NPEV diagnoses were found in MERIN. The fact that the same NPEV genotypes which caused outbreaks across Germany in the same years that they predominated in MERIN indicate that our findings reflect the situation of circulating NPEV genotypes in Germany to a certain extent.

The third most frequent genotype in the MERIN database was coxsackie B, which is one of the most common genotypes causing neonatal infections in Europe [36].

Only one patient in MERIN was diagnosed with enterovirus C infection, but this isolate could not be further typed. Compared with other European countries such as the United Kingdom (UK), France, Belgium and Spain [25,37], occurrence of enterovirus C and D infection (especially enterovirus D68) is still rare in Germany [18,34,37,38]. Since 2017, the Laboratory Network for Enterovirus Diagnostic in Germany has advised that respiratory material from patients with AFP symptoms be tested to detect enterovirus D68 [18,38]. Respiratory specimens are occasionally submitted to MERIN, but enterovirus D was not found.

No polioviruses were detected in MERIN. Testing stools from patients with AFP for poliovirus is the gold standard for polio surveillance [18]. Surveillance of AFP was implemented in Germany in 1998 but failed to reach WHO sensitivity criteria [18]. By investigating patients with meningitis, encephalitis or polio-like symptoms, various NPEVs were detected in MERIN but not polioviruses. Therefore, MERIN helps to document the polio-free status of Lower Saxony and Bremen and can be considered to be an alternative surveillance system.

Proportions of detected pathogens as well as the number of patients with samples submitted to MERIN varied considerably over time. Interestingly, in contrast to Völk et al. [39] who report fewer hospitalisations due to meningitis during the COVID-19 pandemic, the number of MERIN patients did not decrease as strongly as detected pathogens decreased in 2020 and 2021. Presumably, a large proportion of aseptic meningitis and encephalitis cases are of non-infectious origin or the underlying aetiological source remains unidentified [9]. The fact that submissions to MERIN did not greatly decrease during the COVID-19 pandemic suggests a high level of satisfaction among treating physicians. Even during a pandemic, the benefits of rapid and cost-free diagnostics outweighed additional workload described by Łuczyńska et al. [14].

Aside from MERIN’s initial year, the lowest absolute numbers of detected pathogens were observed in 2020 and 2021. This was presumably due to the reduced overall circulation of pathogens during the COVID-19 pandemic [39,40] as a result of non-pharmaceutical interventions such as social distancing, mask wearing in public and increased general hygiene measures. Furthermore, German schools and daycare centres were closed for extended periods during the winter months and not re-opened until late spring in both 2020 and 2021 [40,41]. In 2023, the number of patients with samples submitted to MERIN almost reached pre-pandemic levels, with echovirus 11 being the predominant NPEV genotype. Compared with Kim et al. [23] who reported a decrease in overall NPEV detection from an average of 43.0% to 5.3% during the COVID-19 pandemic, numbers remained much more stable in MERIN and proportions of detected NPEV dropped from an average of 56.9% (2,372/4,172) to 40.2% (43/107) in 2020 and 31.8% (34/107) in 2021.

Both patient numbers and detected NPEVs increased during the summer months, but seasonal patterns were less pronounced during the COVID-19 pandemic years of 2020 and 2021. Increased hospitalisation rates in summer and autumn due to viral meningitis have been previously described [6] and it is well known that NPEV infections peak in summer months [1,9,20]. Reasons for seasonal patterns are still not fully understood and suspected to be either due to changes in human behaviour or increased viral infectiousness favoured by higher temperatures, higher humidity, or both [42]. A fitted regression model confirmed that MERIN data capture seasonality (in pathogen detection and NPEV detection) reasonably well (as shown in Supplement S5), indicating the surveillance system is stable. The years 2003–2006 were not included in the model to avoid potential bias due to strong variation in patient numbers in the initial phase of the surveillance.

As with any surveillance system, MERIN has limitations. Hospitals and clinics, mostly paediatric wards, in Lower Saxony and Bremen participate voluntarily and submit samples from ca 75% of their eligible patients to MERIN [14]. Due to possible selection biases (e.g. voluntary reporting, sampling bias, no strict case definition), our findings cannot be considered representative for the whole population of Lower Saxony and Bremen. However, they do, to some extent, represent hospitalised patients, mostly children, with suspected aseptic meningitis, encephalitis or AFP in Lower Saxony and Bremen. Biases from recruiting or social desirability are less likely, as samples are submitted to MERIN based on clinical symptoms. As the NLGA is the primary investigator, the risk of data loss during data or sample forwarding processes from primary laboratories to national reference laboratories [30] does not apply to MERIN.

Another limitation is that the presented analyses are based on patient outcome and therefore diagnostic results of individual sample specimens are not given. Since physicians submit different sample numbers and material combinations per patient based on individual medical decisions, comparisons are difficult. To minimise investigator bias, the final diagnoses recorded in the MERIN database should be compared with the diagnoses of the treating physicians. A further limitation is that the treating physicians’ final diagnoses were reported back to the NLGA too sporadically to provide useful comparison [14], which should be improved in the future.

Aside from virus cultivation on cells, which to some extent allows for a less directional diagnostic approach, diagnostic outcomes in MERIN are determined by the diagnostic methods employed. The laboratory diagnostic panels offered, as well as surveillance system data collection, need constant evaluation for potential updating [29]. The present overview of the detected pathogen spectrum helps to refine the offered diagnostic panel by identifying redundant pathogens that could be omitted from testing. It also contributes to a greater understanding of pathogen circulation in general. Furthermore, outlining MERIN’s procedures serve as a basis for future evaluations of the surveillance system, and the findings of such evaluations should be published regularly to increase visibility and accessibility for a larger professional audience which could attract additional clinics and increase representativity in the future.

## Conclusion

Despite the mentioned limitations, MERIN successfully fulfils several aims. Firstly, MERIN ensures timely and reliable differential diagnostic clarification for each patient, facilitating individual treatment and case management.

Secondly, MERIN elucidates the spectrum and seasonal occurrence of pathogens causing symptoms of aseptic meningitis, encephalitis or AFP, following the former statutory reporting obligation. In contrast to most EV surveillance systems, where only pre-selected samples of EV are examined, confirmed EV diagnoses are not a prerequisite for inclusion into MERIN, which allows for the detection of various pathogens.

Thirdly, MERIN provides the required information and case numbers to document the polio-free status of Lower-Saxony and Bremen and contributes to national polio surveillance. Using additional clinical symptoms for admission, MERIN is an alternative approach to AFP surveillance that could be used in countries that have been polio-free for many years.

## Data Availability

All data are presented in the manuscript and supported by supplementary material.

## Supplementary Data

681000 Supplementary Material
