# Supplementary material for: The Meningitis and Encephalitis Registry of Lower Saxony, Germany (MERIN) – design and main results of circulating neurotropic pathogen surveillance, 2003 to 2023
Source: Euro Surveill. 2026 Feb 12;31(6):2500625. doi: 10.2807/1560-7917.ES.2026.31.6.2500625 (PMC12905527; doi:10.2807/1560-7917.ES.2026.31.6.2500625)
Supplement: Supplementary Material [file 25-00625_WOLLENWEBER_Supplement.pdf]

Supplementary material

This supplementary material is hosted by Eurosurveillance as supporting information alongside the article "The Meningitis and Encephalitis Registry of Lower Saxony, Germany (MERIN) – design and main results of circulating neurotropic pathogen surveillance, 2003 to 2023", on behalf of the authors, who remain responsible for the accuracy and appropriateness of the content. The same standards for ethics, copyright, attributions and permissions as for the article apply. Supplements are not edited by Eurosurveillance and the journal is not responsible for the maintenance of any links or email addresses provided therein.

Supplement S1: Overview of the MERIN set-up (flowchart)

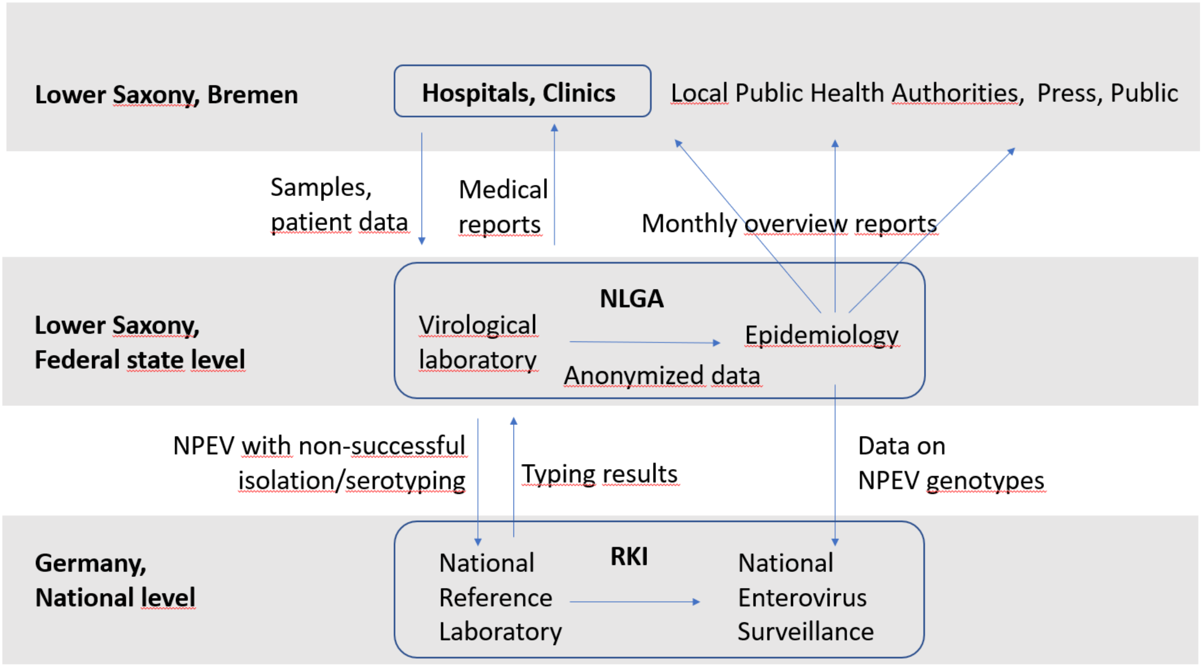

Figure I: Flowchart representing the MERIN (Meningitis and Encephalitis Registry in Lower Saxony) set-up, Public Health Agency of Lower Saxony (NLGA), Hannover, Germany, 2025

Supplement S2: Additional information on the federal public health system, Germany

All 16 German federal states have their own constitution and are largely autonomous in terms of their internal organization. Lower Saxony hosts a population of about eight million inhabitants, whereas the federal state of Bremen has about 700,000 inhabitants and consists of two cities that form an enclave in Lower Saxony[1]. The public health agency of Lower Saxony (Niedersächsisches Landesgesundheitsamt (NLGA)) is a public health institution on federal state level and a subordinate of the Ministry of Health of Lower Saxony's government[2]. The NLGA advises political stakeholders, local health departments and health care institutions and hosts several laboratories tasked with water hygiene investigations, microbiological analyses as well as environmental medicine and hospital hygiene analyses on behalf of public health services. Between 1998 and 2010 the NLGA hosted the National Commission for Polio Eradication in the epidemiological department. MERIN's expenses are covered by the NLGA budget, provided by the federal Ministry of Health. There is also financial compensation for contributions to the national Enterovirus Surveillance via the national Ministry of Health.

Supplement S3: Additional information on the recruiting of MERIN participants

In 2003, hospitals and clinics with paediatric, neurological or internal medicine wards in the German federal state of Lower Saxony that were listed in the German hospital directory[3] were contacted via mail and invited to participate in MERIN and submit samples from patients with aseptic meningitis, encephalitis or polio-like symptoms to the laboratory of the NLGA for charge-free diagnostics. The same offer was made in 2011 towards hospitals and clinics in the federal state of Bremen.

An assessment to determine active contributors to MERIN was conducted in 2021 and identified 32 hospitals (29 from Lower Saxony, 3 from Bremen) with a total of 33 wards (24 paediatric, 7 neurological and 2 internal wards) that submitted samples of at least one patient over the past two years prior to the survey[4,5].

In 2025, the offer to submit samples for free-of-charge diagnostics was renewed by contacting all 31 paediatric, 36 neurological and 102 internal wards of 218 hospitals in Lower Saxony and 4 paediatric, 5 neurological and 7 internal medicine wards of 8 eligible hospitals in Bremen that were listed in the current version of the German hospital directory via mail. Contributing hospitals are not formally registered, but samples and submission forms are checked upon arrival at the NLGA laboratory and analyses are only performed if admission criteria are met.

---

[1] Fazit Communication GmbH on behalf of the Foreign Office. Die 16 Bundesländer. [The 16 federal states.] Frankfurt /Main; 2025. German. Available from: https://www.deutschland.de/de/topic/politik/deutschland-europa/laender

[2] Niedersächsisches Landesgesundheitsamt (NLGA). Aufgaben, Struktur und Geschichte des Landesgesundheitsamts. [Responsibilities, structure and history of the Public Health Agency.] Hannover: NLGA; [Accessed: 10.01.2026]. German. Available from: https://www.nlga.niedersachsen.de/aufgaben-struktur/aufgaben-struktur-und-geschichte-des-landesgesundheitsamtes-203041.html

[3] Deutsches Krankenhaus TrustCenter und Informationsverarbeitung GmbH (DKTIG). Deutsches Krankenhausverzeichnis. [German hospital directory.] Leipzig; 2026. German Available from: https://www.deutsches-krankenhaus-verzeichnis.de/app/suche

[4] Łuczyńska A, Beyrer K, Holle I, Baillot A, Monazahian M, Dreesman J, et al. Evaluation of 17 years of MERIN (Meningitis and Encephalitis register in Lower Saxony, Germany) surveillance system: participants acceptability survey, completeness and timeliness of data. BMC Health Serv Res. 2024;24(1):59. http://dx.doi.org/10.1186/s12913-023-10482-y PMID:38212779

[5] Łuczyńska et al., unpublished data

Supplement S4:
Laboratory workflows at the NLGA (for different sample materials)

S4a: Laboratory workflow for cerebrospinal fluid (CSF) samples

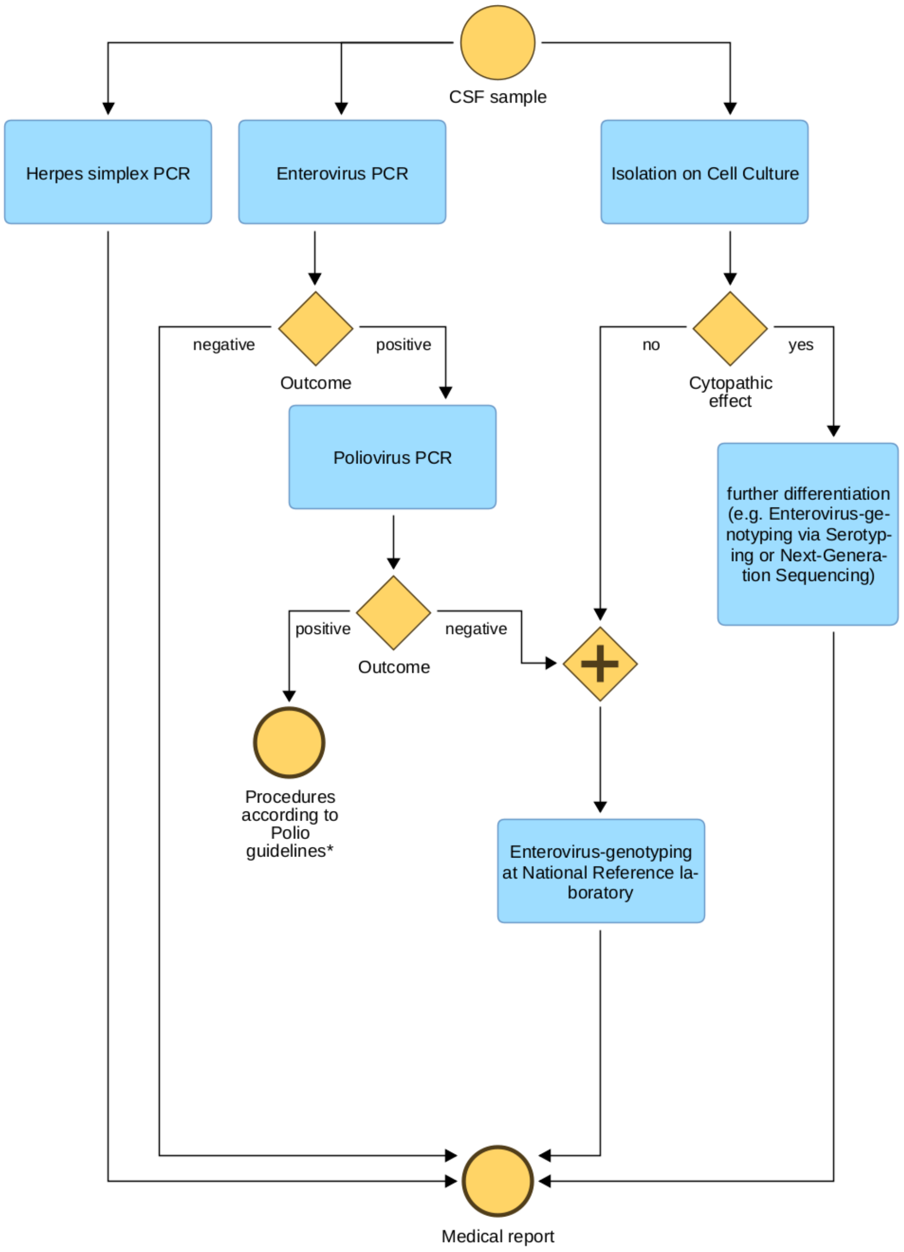

*Procedures according to Polio guidelines are outside the scope of MERIN.

Figure II: Laboratory workflow and diagnostic procedures for cerebrospinal fluid (CSF) samples from patients investigated within the framework of MERIN (Meningitis and Encephalitis Registry in Lower Saxony, 2003-2023) at the Public Health Agency of Lower Saxony (NLGA), Hannover, Germany, 2025.

S4b: Laboratory workflow for stool samples

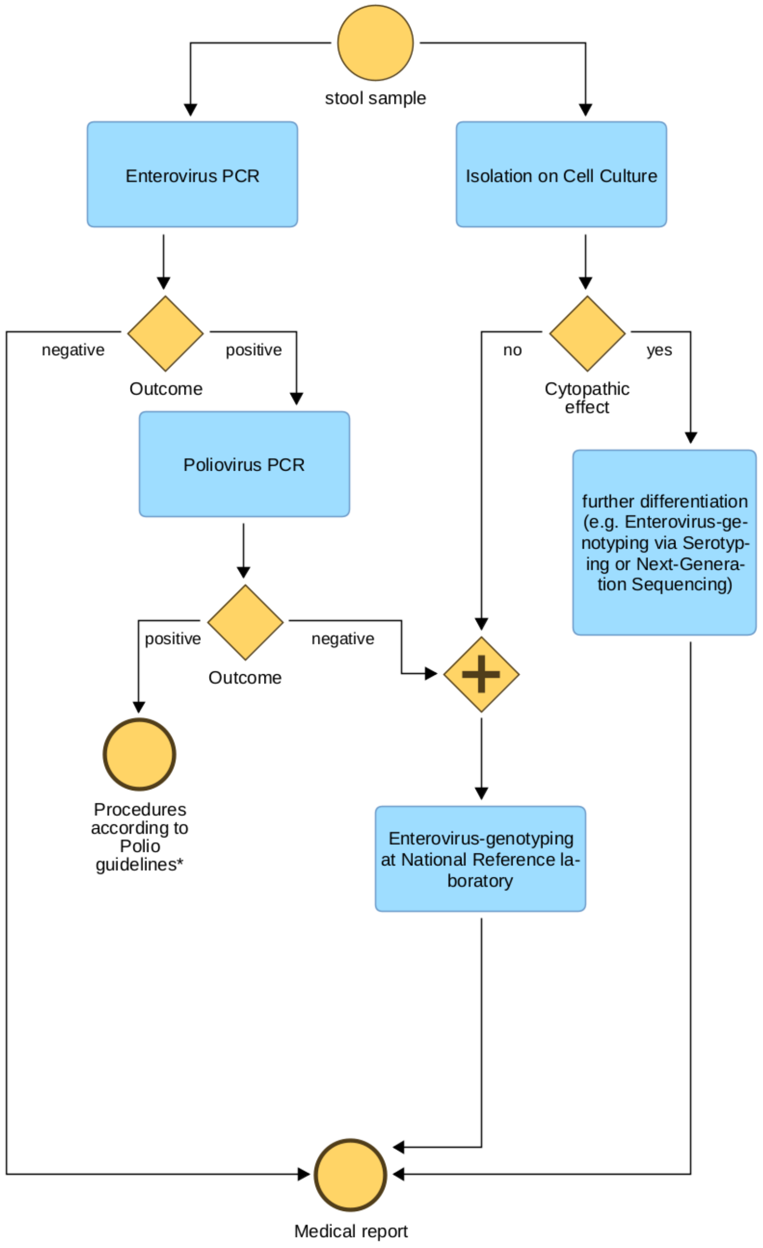

*Procedures according to Polio guidelines are outside the scope of MERIN.

Figure III: Laboratory workflow and diagnostic procedures for stool samples from patients investigated within the framework of MERIN (Meningitis and Encephalitis Registry in Lower Saxony, 2003-2023) at the Public Health Agency of Lower Saxony (NLGA), Hannover, Germany, 2025.

S4c: Laboratory workflow for respiratory swab samples

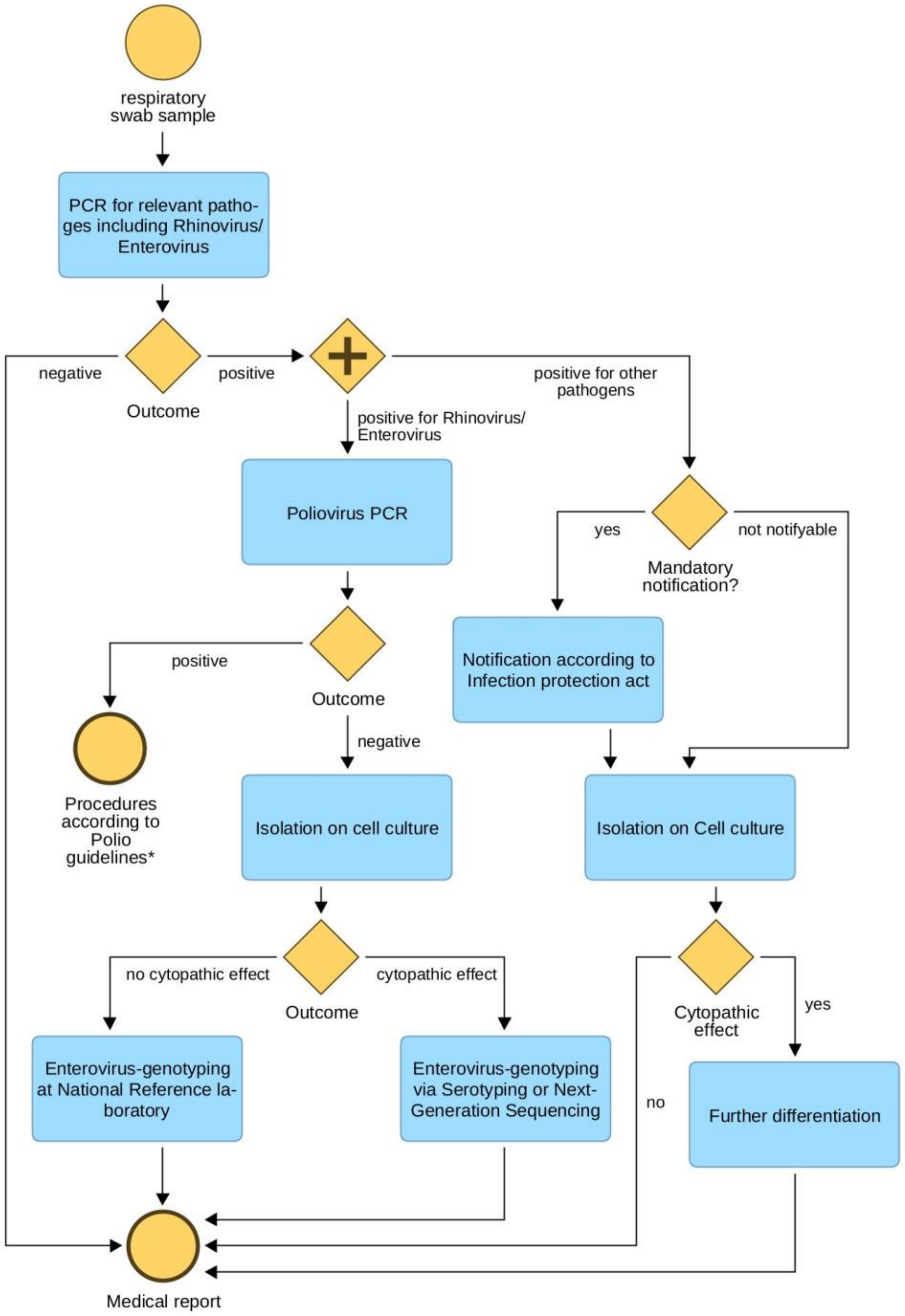

*Procedures according to Polio guidelines are outside the scope of MERIN.

Figure IV: Laboratory workflow and diagnostic procedures for respiratory swab samples from patients investigated within the framework of MERIN (Meningitis and Encephalitis Registry in Lower Saxony, 2003-2023) at the Public Health Agency of Lower Saxony (NLGA), Hannover, Germany, 2025.

S4d: Laboratory workflow for blood and serum samples

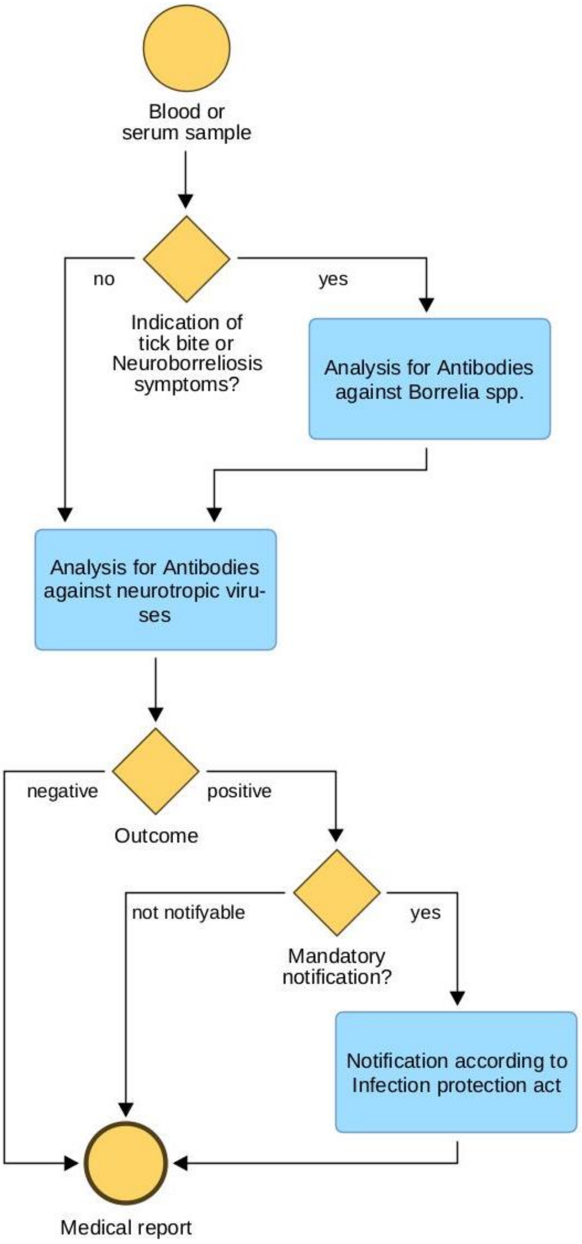

*Procedures according to Polio guidelines are outside the scope of MERIN.

Figure V: Laboratory workflow and diagnostic procedures for blood and serum samples from patients investigated within the framework of MERIN (Meningitis and Encephalitis Registry in Lower Saxony, 2003-2023) at the Public Health Agency of Lower Saxony (NLGA), Hannover, Germany, 2025.

Supplement S5:
Detailed results of Poisson Regression model investigating seasonality

Table I: Poisson Regression model including all MERIN-patients with pathogens detected (any), 2007-2023, including terms for COVID-19-pandemic years (2020 & 2021) and outbreak years of 2008 and 2013, investigating seasonality. Effect sizes are determined as the corresponding exponentiated regression coefficients. AIC= 1423.3

**Coefficients**	**Effect size**	**95% CI**	**p-value**
Intercept	15.298	14.673 – 15.951	<0.001
sine	0.495	0.471 – 0.521	<0.001
cosine	0.705	0.672 – 0.740	<0.001
COVID-19 pandemic	0.503	0.437 – 0.578	<0.001
Outbreak years	1.416	1.297 – 1.546	<0.001

Table II: Poisson Regression model including all MERIN-patients with NPEV detected, 2007-2023, including terms for COVID-19-pandemic years (2020 & 2021) and outbreak years of 2008 and 2013, investigating seasonality. Effect sizes are determined as the corresponding exponentiated regression coefficients. AIC= 1197.3

**Coefficients**	**Effect size**	**95% CI**	**p-value**
Intercept	7.451	6.993 – 7.939	<0.001
sine	0.357	0.330 – 0.386	<0.001
cosine	0.659	0.616 – 0.705	<0.001
COVID-19 pandemic	0.330	0.262 – 0.415	<0.001
Outbreak years	1.834	1.645 – 2.044	<0.001

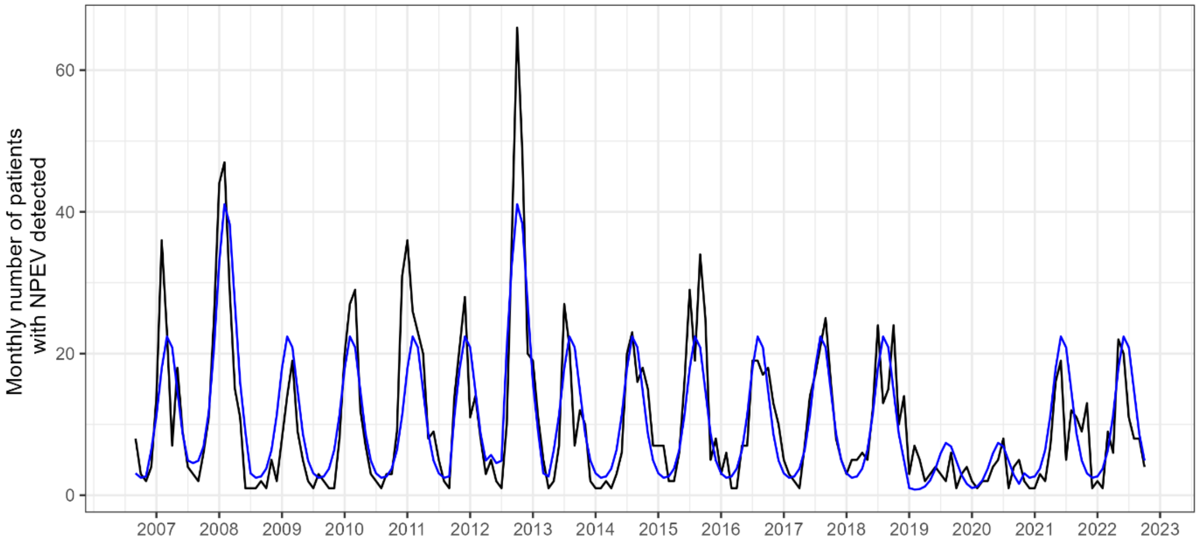

Figure VI: Seasonality of NPEV (non-polio enteroviruses) detected in MERIN (Meningitis- and Encephalitis Registry in Lower Saxony) between 2007 and 2023 (black line) compared to predicted detections modelled using Poisson regression (blue line) with Akaike Information Criterion (AIC) of 1197.3 for NPEV; Lower Saxony and Bremen[a], Germany, 2007-2023[b]

[a] Data from Bremen were added to Lower-Saxony data from 2011 onwards

[b] The years 2003–2006 were not included in the Poisson regression model to avoid potential bias due to strong variation in patient numbers in the initial phase of the surveillance.
